# DNA methylome analysis identifies epigenetic silencing of FHIT as a determining factor for radiosensitivity in oral cancer: an outcome-predicting and treatment-implicating study

**DOI:** 10.18632/oncotarget.2821

**Published:** 2014-11-25

**Authors:** Hon-Yi Lin, Shih-Kai Hung, Moon-Sing Lee, Wen-Yen Chiou, Tze-Ta Huang, Chih-En Tseng, Liang-Yu Shih, Ru-Inn Lin, Jora M.J. Lin, Yi-Hui Lai, Chia-Bin Chang, Feng-Chun Hsu, Liang-Cheng Chen, Shiang-Jiun Tsai, Yu-Chieh Su, Szu-Chi Li, Hung-Chih Lai, Wen-Lin Hsu, Dai-Wei Liu, Chien-Kuo Tai, Shu-Fen Wu, Michael W.Y. Chan

**Affiliations:** ^1^ Department of Radiation Oncology, Buddhist Dalin Tzu Chi General Hospital, Taiwan, ROC; ^2^ Department of Oral and Maxillofacial Surgery, Buddhist Dalin Tzu Chi General Hospital, Taiwan, ROC; ^3^ Department of Anatomic Pathology, Buddhist Dalin Tzu Chi General Hospital, Taiwan, ROC; ^4^ Department of Hematology-Oncology, Buddhist Dalin Tzu Chi General Hospital, Taiwan, ROC; ^5^ Department of Radiation Oncology, Buddhist Tzu Chi General Hospital, Hualien, Taiwan, ROC; ^6^ School of Medicine, Tzu Chi University, Hualien, Taiwan, ROC; ^7^ Institute of Oral Medicine, National Cheng Kung University, Tainan, Taiwan, ROC; ^8^ Institute of Molecular Biology, National Chung Cheng University, Min-Hsiung, Chia-Yi, Taiwan, ROC; ^9^ Department of Life Science, National Chung Cheng University, Min-Hsiung, Chia-Yi, Taiwan, ROC; ^10^ Human Epigenomics Center, National Chung Cheng University, Min-Hsiung, Chia-Yi, Taiwan, ROC

**Keywords:** Epigenetics, FHIT, oral cancer, radiotherapy, resistance

## Abstract

Radioresistance is still an emerging problem for radiotherapy of oral cancer. Aberrant epigenetic alterations play an important role in cancer development, yet the role of such alterations in radioresistance of oral cancer is not fully explored. Using a methylation microarray, we identified promoter hypermethylation of *FHIT* (fragile histidine triad) in radioresistant OML1-R cells, established from hypo-fractionated irradiation of parental OML1 radiosensitive oral cancer cells. Further analysis confirmed that transcriptional repression of *FHIT was* due to promoter hypermethylation, H3K27me3 and overexpression of methyltransferase *EZH2* in OML1-R cells. Epigenetic interventions or depletion of *EZH2* restored *FHIT* expression. Ectopic expression of *FHIT* inhibited tumor growth in both *in vitro* and *in vivo* models, while also resensitizing radioresistant cancer cells to irradiation, by restoring Chk2 phosphorylation and G2/M arrest. Clinically, promoter hypermethylation of *FHIT* inversely correlated with its expression and independently predicted both locoregional control and overall survival in 40 match-paired oral cancer patient samples. Further *in vivo* therapeutic experiments confirmed that inhibition of DNA methylation significantly resensitized radioresistant oral cancer cell xenograft tumors. These results show that epigenetic silencing of *FHIT* contributes partially to radioresistance and predicts clinical outcomes in irradiated oral cancer. The radiosensitizing effect of epigenetic interventions warrants further clinical investigation.

## INTRODUCTION

Radiotherapy is an important modality for the treatment of oral cancer. After radical surgery, post-operative radiotherapy (with or without chemotherapy) is recommended for patients with pathologically adverse features, such as pT3-4, pN1-3, extracapsular nodal extension, and positive surgical margins [1]. However, a major obstacle is the development of radioresistant cancer cells, eventually leading to locoregional recurrences, and the molecular mechanism that underlies radioresistance is not fully understood.

Unlike genetic events, epigenetic modifications do not involve changes of DNA sequences, but do have profound effects on gene promoter activity [2-4]. Two types of epigenetic modifications are crucial in cancers. First, DNA methylation of promoter “CpG islands” of tumor suppressor genes, resulting in their transcriptional repression, is frequently observed in cancers [5-7]. Second, specific post-translational modifications of chromatin proteins, such as EZH2-mediated trimethylation of histone 3 lysine 27 (H3K27me3), are known transcriptional silencing gene “marks” [8-10]. Interestingly, EZH2-bound region of the genes marked by H3K27me3 are able to recruit DNA methyltransferases (DNMTs) for DNA methylation, also resulting in epigenetic silencing of tumor suppressor genes [11].

We and others have previously demonstrated that aberrant promoter hypermethylation of tumor suppressor genes is an important cancer-specific event useful for predicting prognosis in several human cancers [12-15]. For example, we have previously identified promoter hypermethylation of *DAPK* to be predictive of locoregional control in head and neck cancers [16]. However the role of aberrant epigenetic alterations in radioresistance of oral cancer is not fully explored.

In the current study, we established an *in vitro* model to investigate the role of aberrant epigenetic modifications in the development of oral cancer radioresistance. Using a methylation microarray, we show that promoter hypermethylation of *Fragile histidine triad (FHIT)* facilitates radioresistance after massive irradiation in oral cancer cells, and that this event has *in vitro* and *in vivo* prognostic value for demarcating possible radio-resensitization of this deadly disease by epigenetic interventions.

## RESULTS

### Differential methylation analysis between OML1-P and OML1-R cells

**Figure 1:**
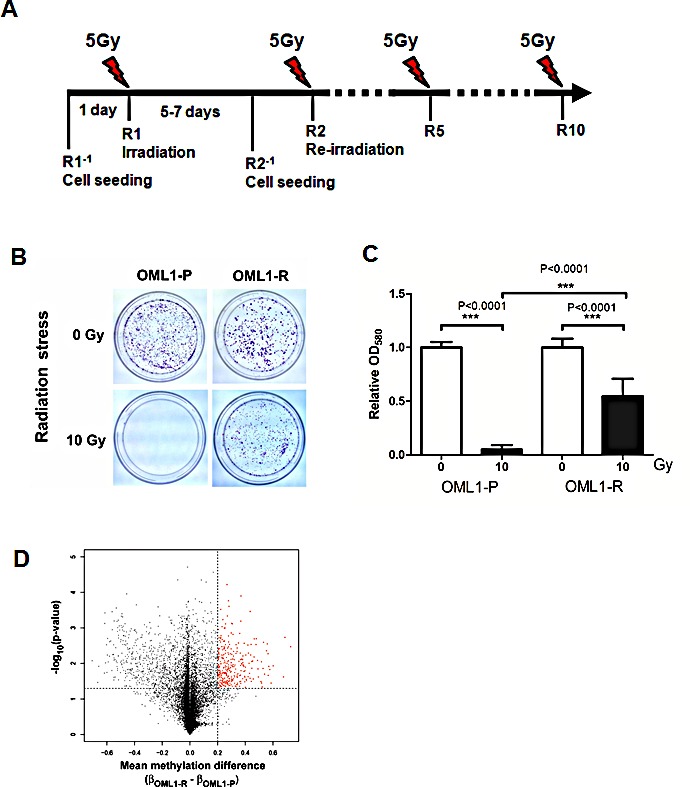
Establishment of a radioresistant oral cancer cell subline and its differential methylation profile (A) Timeline schema for establishing a radioresistant oral cancer cell subline. One day before irradiation (R1^−1^, R2^−1^…etc), cells were seeded onto 10-cm cell culture plates. On the following day (R1, R2…R10), a fraction of 5-Gy irradiation was delivered using a 6-MV linear accelerator. The cells were allowed to recover for 5-7 days before another round of irradiation. This process was repeated for up to 10 fractions (R10) until a total dose of 50 Gy was obtained. The irradiations from R2 to R10 are indicated by dash line. (B) Radiation stress test in parental OML1-P and radioresistant OML1-R cells using a single 10-Gy irradiation. Cells were allowed to recover for several days and then stained with 0.4% crystal violet (C) Quantitative analysis of the radiation stress test described in B. Stained cells were lysed and measured by a spectrophotometer, and the relative number of cells were expressed as OD_580_. Data are expressed as means ± SD (n=3). (D) Volcano plot showing methylation differences and P values of each probe between OML1-R and their parental OML1-P cells. A total of 24,000 gene loci were compared. Of these, 330 probes (red) demonstrated an increased β value ≥ 0.2 and P <0.05, in OML1-R cells.

To explore the role of epigenetic modifications in the onset of radioresistant oral cancer, we developed a radioresistant oral cancer cell (OML1-R) subline, from parental OML1-P cell, using a hypo-fractionated irradiation protocol (5Gy by 10 fractions; Fig. 1A). A single test fraction of 10-Gy irradiation confirmed the established radioresistance of OML1-R cells, as compared to the parental cells (*P* < 0.0001; Fig. 1B,C).

To identify genes differentially methylated in radioresistant cells, OML1-R and the parental cells were compared by methylation analysis using Illumium 27K methylation BeadChip microarrays. Of approximately 300 probes found to be significantly hypermethylated in OML1-R cells (Fig. 1D, red spots), and filtering for probes with initial β values of <0.5 in the parental cells, 180 probes were found significantly hypermethylated (Supplementary Table S2). Subjecting this set of differentially methylated genes to ontology analysis by DAVID [17] revealed several significantly enriched biological processes (Supplementary Table S3).

Aberrant DNA methylation has been previously reported in cellular non-response to ionizing radiation, particularly for genes involved in cell cycle control, DNA repair, and apoptosis [18]. In this regard, genes significantly enriched in the corresponding biological processes (purine nucleoside metabolic process and DNA metabolic process), based on our gene ontology analysis, were further screened. One of the targets, *FHIT*, a gene that regulates G2/M checkpoint and apoptosis was then selected for further analysis. *FHIT* was also selected based on its location in a fragile chromosome site (3p13.2) that would likely be damaged by ionizing irradiation [19].

### *FHIT* is epigenetically silenced in OML1-R cells

**Figure 2:**
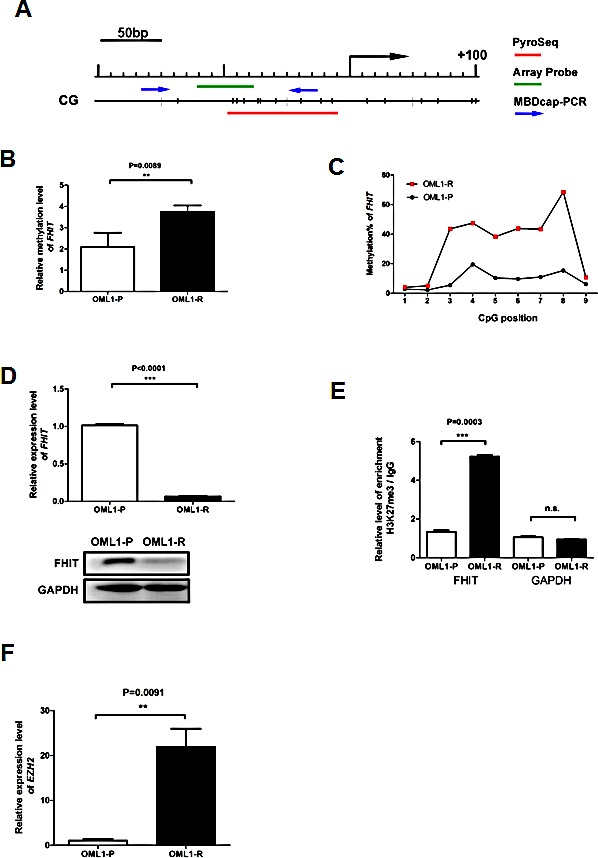
Validation of promoter methylation and FHIT expression in oral cancer cells (A) Schematic diagram depicting the position of CG sites in the promoter region of *FHIT*. The red line represents the region subjected to bisulphite pyrosequencing, containing 9 CpG sites. The location of the microarray probe (green), and primers for MBDcap-PCR (blue arrows) are also indicated. The methylation level of *FHIT* was determined by (B) MBDcap-PCR and (C) bisulphite pyrosequencing. (D) Expression of *FHIT* in the radio-sensitive vs. –resistant cells was also determined by qRT-PCR (upper panel) and Western blot analysis (lower panel). (E) ChIP assays were performed with antibodies against trimethyl-H3-K27 (H3K27me3) in the promoter regions of *FHIT* and GAPDH (as control) in OML1-P and OML1-R cells. The relative level of enrichment was determined by real-time PCR and normalized to the Ct value of IgG-pulled-down DNA alone. Higher levels of H3K27me3 were observed in OML1-R cells. (F) Expression of *EZH2* in OML1-P and OML1-R was determined by qRT-PCR. Data in the histogram are expressed as means ± SD (n=3).

To validate our microarray result, we performed methylated-binding DNA (MBD) capture coupled to real time PCR (MBDcap-PCR), in addition to bisulphite pyrosequencing, of the promoter region of *FHIT* (Fig. 2A). Both MBDcap-PCR (Fig. 2B) and bisulphite pyrosequcening (Fig. 2C) confirmed higher *FHIT* promoter methylation in OML1-R than in OML1-P cells. More importantly, *FHIT* promoter hypermethylation associated with down-regulation of its mRNA and protein (Fig. 2D). These phenomenon may be attributed to an increased expression of *DNMT3a*, *3b* but not *DNMT 1* in OML1-R cells (Fig. S2).

We also conducted H3K27me3 ChIP-PCR to examine the histone chromatin status of the promoter region of *FHIT* in OML1-R and the parental cells. As expected, OML1-R cells possessed higher H3K27me3 levels around the promoter region of the *FHIT* gene (Fig. 2E). The enrichment of this repressive histone mark may be due to overexpression of the histone methyltransferase, *EZH2* in OML-1R cells (Fig. 2F).

**Figure 3:**
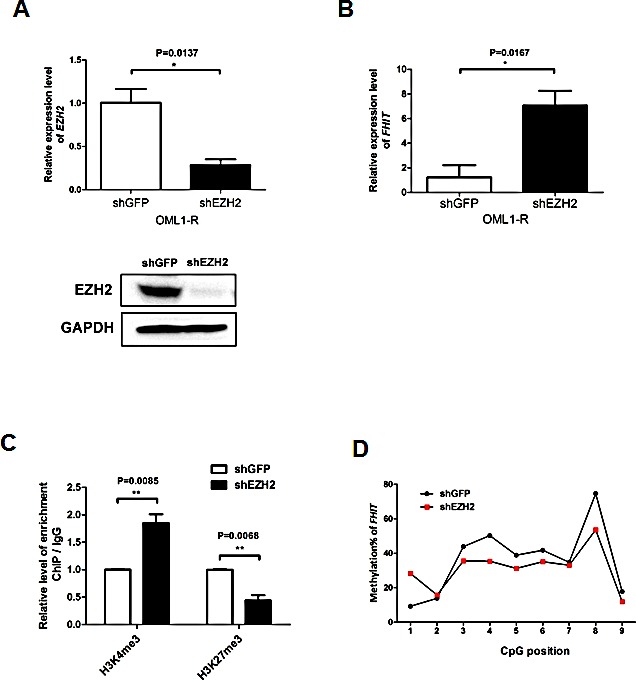
The role of EZH2 in the epigenetic silencing of *FHIT* OML1-R cells were infected with shRNA against EZH2 or GFP (control). (A) Expression of *EZH2* in the infected cells were determined by qRT-PCR (upper panel) and Western blot analysis (lower panel). (B) Expression level of *FHIT* was determined by qRT-PCR in control and EZH2-depleted OML1-R cells. Histone modifications (H3K4me3 and H3K27me3) and promoter methylation of *FHIT* in the control and EZH2-depleted cells were determined by ChIP-qPCR and bisulphite pyrosequencing respectively. Interestingly, depletion of *EZH2* reversed histone modifications (C) and reduced promoter methylation (D) of *FHIT* in EZH2-depleted OML1-R cells.

EZH2 is a key component of the Polycomb repressive complex 2 (PRC2) and is involved in transcriptional repression [20]. Previous studies demonstrated that EZH2-mediated H3K27me3 correlates tightly with DNA methylation [11, 21-22] or at least in a regional-dependent manner [23]. However, contradictory evidences also suggested that H3K27me3 and DNA methylation are mutually exclusive [24-25]. To examine the role of EZH2 in the epigenetic silencing of *FHIT*, we depleted EZH2 in OML1-R cells (Fig 3A). As expected, lentiviral knock-down of *EZH2* resulted in a re-expression of *FHIT* in OML1-R cells (Fig 3B). This re-expression is accompanied by a relaxation of chromatin as demonstrated by about 2-fold increase of H3K4me3 and 2-fold decrease of H3K27me3 in the promoter region of *FHIT* (Fig 3C). Importantly, EZH2-depleted cells also showed a 10%-decrease of *FHIT* promoter methylation as demonstrated by bisulphite pyrosequencing (Fig 3D). These results suggested that promoter methylation of *FHIT* is partially controlled by EZH2 in the promoter region of *FHIT*.

### Epigenetic drug treatment restores *FHIT* expression in OML1-R cells

**Figure 4:**
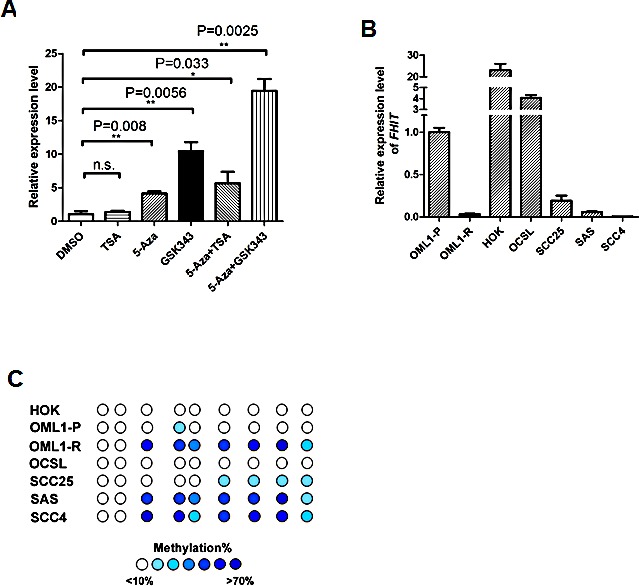
*FHIT* is epigenetically silenced in oral cancer cells (A) Relative expression of *FHIT* in OML1-R cells after epigenetic interventions. OML1-R cells were treated with various drugs as indicated. The expression level of *FHIT* was then determined by qRT-PCR. The highest reexpression of *FHIT* was observed in co-treatment with 5-Aza and EZH2 inhibitor, GSK343. (B) Relative expression of *FHIT* in HOK (human oral keratinocyte), OML1 cells and different oral cancer cell lines (OCSL, SCC25, SAS, SCC4) was determined by qRT-PCR, using the expression level of *FHIT* in OML1-P cells as a reference of 1.0. The highest *FHIT* expression levels were observed in HOK cells. *FHIT* promoter methylation levels in these cells were determined by bisulphite sequencing, as shown in (C). The percent methylation of each CpG site is indicated by the intensity of blue. Data in the histogram are expressed as means ± SD (n=3).

To further investigate whether epigenetic derepressors might reverse *FHIT* silencing, we found that treatment of OML1-R cells with a DNMT inhibitor (5-aza-2′-deoxycytidine, 5-Aza) alone, but not an HDAC inhibitor (TSA) alone, could restore *FHIT* expression (Fig. 4A), while combination 5-Aza/TSA treatment resulted in additive effect of *FHIT* re-expression. Interestingly, treatment with an EZH2 inhibitor (GSK343), which specifically inhibits H3K27me3 [26], elicited robust reactivation of *FHIT*. Remarkably, combination treatment of 5-Aza and GSK343 resulted in the highest level of *FHIT* re-expression (Fig. 4A).

### *FHIT* is epigenetically silenced in a panel of oral cancer cell lines

We then explored the correlation between *FHIT* expression and promoter methylation in a primary human oral keratinocyte (HOK) and four other oral cancer cell lines (OCSL, SCC25, SAS, and SCC4). As expected, similar to OML1-R cells, *FHIT* expression (Fig. 4B) demonstrated a tight inverse correlation with promoter methylation of *FHIT* (Fig. 4C) in those cells. Taken together, these results strongly suggest that epigenetic modifications contribute to the process of radioresistance via down-regulation of *FHIT* in oral cancer cells.

### Overexpression of *FHIT* restores radiosensitivity in OML1-R cells

**Figure 5:**
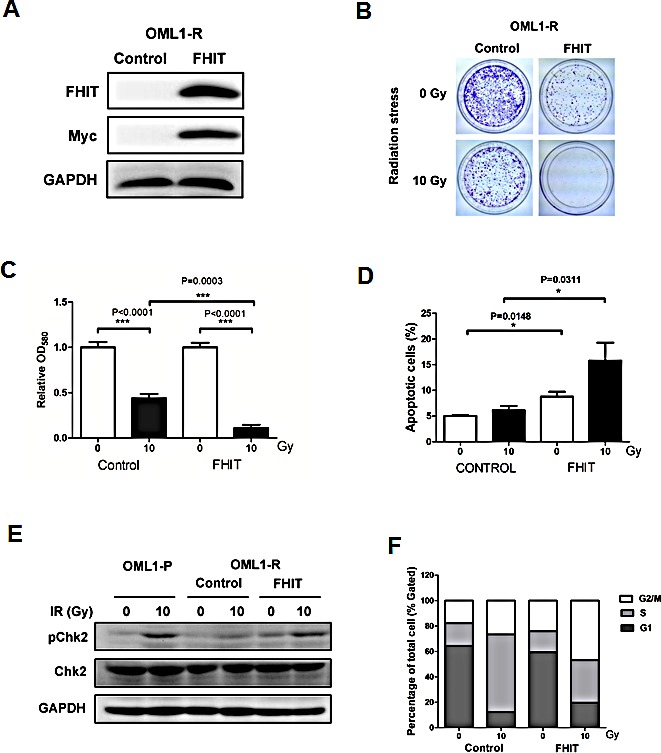
Overexpression of *FHIT* restores radiosensitivity to OML1-R cells (A) OML1-R cells overexpressing an FHIT cDNA Myc-Tag plasmid. Protein levels of FHIT, Myc and GAPDH (loading control) in OML1-R cells overexpressing empty vector (control) or FHIT expression vector (FHIT) were analyzed by Western blot. Strong *FHIT* overexpression was observed in FHIT-overexpressing cells. (B) Radiation stress test in *FHIT*-overexpressing OML1-R cells. Control or *FHIT*-overexpressing OML1-R cells were irradiated with a single 10-Gy irradiation in a 10cm dish. *FHIT*-overexpressing OML1-R cells showed fewer colony numbers than those of control OML1-R cells. The cells were stained with 0.4% crystal violet and the amount of dye measured by a spectrophotometer as shown in (C). (D) Effect of *FHIT* on radiation-induced apoptosis in OML1-R cells. Control or FHIT-overexpressing cells were irradiated with a single 10-Gy irradiation shot. Cells were treated with Annexin V-FITC and propidium iodide (PI). The percentage of apoptotic cells were determined by flow cytometry. Representative flow cytometry results can be found in Figure S3. Overexpression of *FHIT* enhanced radiation-induced apoptosis in OML1-R cells. (E) Protein levels of Chk2, phosphorylated Chk2 (pChk2), and GAPDH (loading control) in OML1-P and *FHIT*-overexpressing OML1-R cells. Overexpression of *FHIT* restored phosphorylation of Chk2 in OML1-R cells. (F) Cells were treated with PI and the percentage of cells at different phase of cell cycle was determined by flow cytometry. Data in histograms are expressed as means ± SD (n=3).

Next, we want to investigate the role of *FHIT* in radiosensitivity. OML1-R cells were transfected with an *FHIT-*overexpressing cDNA myc-tag plasmid (Fig. 5A). Compared to control cells, OML1-R cells overexpressed with *FHIT* formed fewer colonies after a single fraction of 10-Gy irradiation, supporting a role for *FHIT* in radiosensitivity (Fig. 5B,C). Moreover, overexpression of *FHIT* significantly induced apoptosis in OML1-R cells after irradiation (Fig. 5D and S3). Finally, *FHIT* overexpression restored phosphorylation of Chk2 (Fig. 5E), expression of p21 (Fig. S4) and activation of G2/M checkpoint (Fig. 5F) in irradiated OML1-R cells. Taken together, these results suggest that *FHIT* may mediate radiosensitivity by inducing apoptosis and activation of a G2/M checkpoint.

### Expression of *FHIT* is associated with radiosensitivity in oral cancer cells

**Figure 6:**
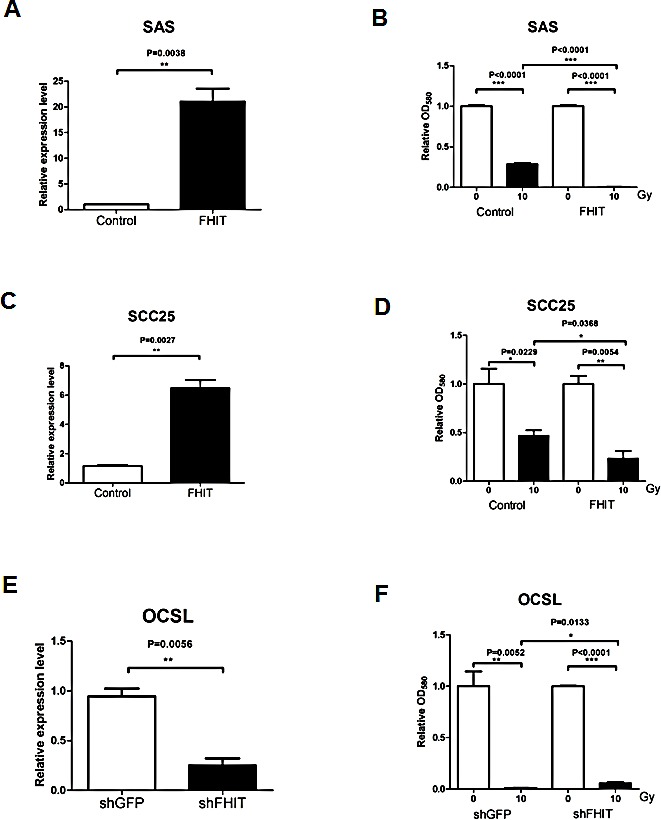
Expression of *FHIT* determines radiosensitivity in oral cancer cells *FHIT* was overexpressed in SAS (A, B) and SCC25 (C, D) oral cancer cells, which both showed low *FHIT* expression. The relative expression level of *FHIT* after transfection was determined by qRT-PCR (A, C). Radiosensitivity of the control and *FHIT*-overexpressing cells was determined by 10-Gy radiation stress test (B, D), as previously mentioned. Overexpression of *FHIT* significantly enhanced the radiosensitivity of both SAS and SCC25 cells. (E) OCSL oral cancer cells were infected with shRNA against *FHIT*, resulting in a significant reduction of *FHIT* expression, as determined by qRT-PCR. (F) Radiation stress test demonstrated a slight reduction of radiosensitivity in *FHIT*-knockdown OSCL cells. Data in histograms are expressed as means ± SD (n=3).

The above experiments demonstrated that epigenetic silencing of *FHIT* may contribute to the radioresistance in OML1-R cells, while restoring *FHIT* expression facilitates radiosensitivity. To avoid the possibility of cell line effects, we investigated the radiosensitivity in another two oral cancer cell lines (SAS, SCC25), both of which endogenously underexpress *FHIT* (Fig. 4B). Interestingly, both SAS and SCC25 cells demonstrated an intrinsic radioresistance (Fig 6B,D, Control), while ectopic *FHIT* expression significantly increased the radiosensitivity in these two cells (Fig. 6A-D).

We also performed a reciprocal experiment of knocking down *FHIT* in OSCL cells, which intrinsically highly express *FHIT* (Fig. 6E). Consistent with a role in radiosensitivity, *FHIT* knockdown slightly enhanced OSCL cell radioresistance (Fig. 6F). These results further suggest a role for *FHIT* in oral cancer radiosensitivity.

### Epigenetic silencing of *FHIT* is correlated with poor clinical outcome in oral cancer patients

**Figure 7:**
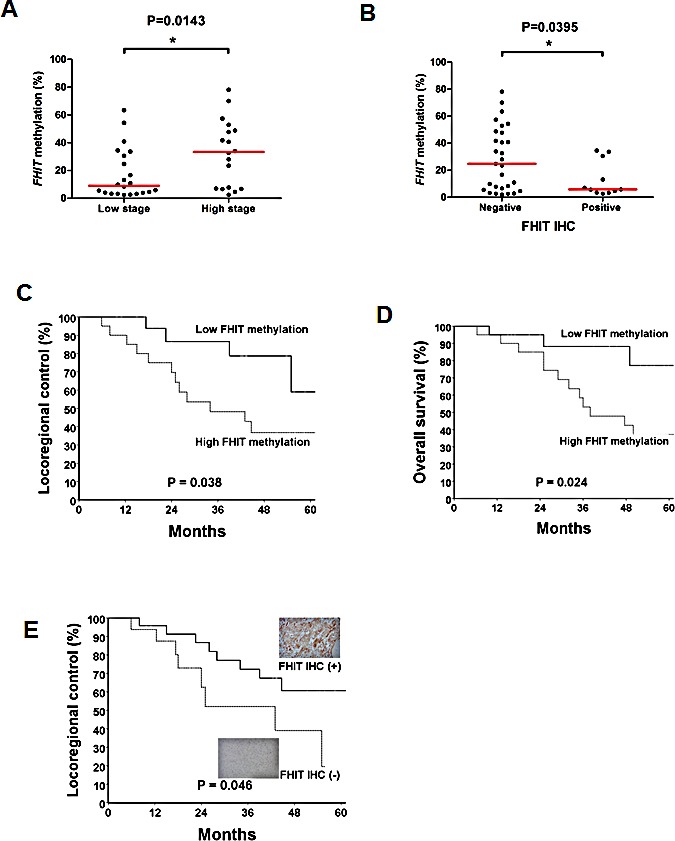
Epigenetic silencing of *FHIT* associates with poor prognosis in oral cancer patients (A) *FHIT* promoter methylation levels in 40 oral cancer patient samples were determined by bisulphite pyrosequencing. Values in dot plots represent average percent methylation of the 9 CpG sites in the *FHIT* promoter from each sample. High-staged patients (IV A/B) demonstrated significantly higher methylation levels of *FHIT* than low-staged patients (non-IVA/B). Horizontal lines represent a median level. (B) Association between expression of *FHIT* and promoter methylation of *FHIT* in oral cancer patient samples. Patients with negative IHC of *FHIT* have significantly higher promoter methylation of FHIT than that of patients with positive IHC of FHIT. Kaplan–Meier analysis for locoregional control (C) and overall survival (D) in oral cancer patients. Patients with higher *FHIT* methylation demonstrated lower locoregional control rates (*P* = 0.038) and shorter overall survival (*P* = 0.024) than patients with lower *FHIT* methylation. (E) Kaplan Meier analysis showing that oral cancer patients with lower FHIT expression exhibited lower locoregional control (*P* = 0.046) rate than that of patients with higher FHIT expression, as determined by immunohistochemistry. The representative images of positive (upper insert) and negative (lower insert) IHC stain of FHIT are shown (400X). The corresponding high resolution images can be found in Fig. S5.

To further examine a clinical role for *FHIT* in oral cancer progression, we investigated its promoter methylation and expression in 40 match-paired, paraffin-embedded oral cancer patient samples (Fig. S1, Table 1 and 2). Results from bisulphite pyrosequencing revealed a higher median methylation level in high-staged patients than that of low-staged patients (*P* = 0.0143; Fig. 7A). Importantly, patients with a negative staining of FHIT demonstrated a significantly higher promoter methylation of *FHIT* than that of patients with positive staining of FHIT (*P* = 0.0395, Fig. 7B). Moreover, patients with a higher *FHIT* methylation (>10%, n=22/40) showed a lower locoregional control rate than that of patients with lower methylation (*P* = 0.038; Fig. 7C). Remarkably, this predicting ability further translated into overall survival (*P* = 0.024; Fig. 7D). To exclude the effect of chemotherapy, we also performed a subgroup analysis in patients treated with post-operative radiotherapy alone (n=19). However, due to the small sample size, we only observed a trend that patients with higher *FHIT* methylation tend to have a 2-fold lower locoregional control rate than that of patients with lower methylation (survival%: high vs low methylation, 33.9% vs 66.7%, *P* = 0.0998). Moreover, consistent with methylation results, patients lacking FHIT expression also showed a lower locoregional control rate than that of patients expressing FHIT (*P* = 0.046; Fig. 7E, Fig. S5).

**Table 1:** Patient characteristics according to distance of pathological surgical margin

	Pathological surgical margin, *n* (%)		Total, *n* (%)	*P*value
	>1 ~ ≤5 mm	>0 ~ ≤1 mm		
**Patient**				
Age*				*0.99*
≤50 years	8 (40.0)	8 (40.0)	16 (40.0)	
>50 years	12 (60.0)	12 (60.0)	24 (60.0)	
Gender*				*0.99*
Female	1 (5.0)	1 (5.0)	2 (5.0)	
Male	19 (95.0)	19 (95.0)	38 (95.0)	
ECOG PS				*0.99*
0-1	17 (85.0)	17 (85.0)	34 (85.0)	
≥2	3 (15.0)	3 (15.0)	6 (15.0)	
Betel nut chewing				*0.67*
No	3 (15.0)	4 (20.0)	7 (17.5)	
Yes	17 (85.0)	16 (80.0)	33 (82.5)	
**Tumor**				
Histology				*0.15*
Grade 1-2	19 (95.0)	16 (80.0)	35 (87.5)	
Grade 3-4	1 (5.0)	4 (20.0)	5 (12.5)	
pT status				*0.18*
pT1-3	16 (80.0)	11 (55.0)	27 (67.5)	
pT4	4 (20.0)	9 (45.0)	13 (32.5)	
pN status				*0.67*
pN0	16 (80.0)	17 (85.0)	33 (82.5)	
pN1-2	4 (20.0)	3 (15.0)	7 (17.5)	
Pathology stage*				*0.99*
non-IVA/B	11 (55.0)	11 (55.0)	22 (55.0)	
IVA/B	9 (45.0)	9 (45.0)	18 (45.0)	
**Treatment**				
Post-OP therapy				*0.21*
RT alone	12 (60.0)	7 (35.0)	19 (47.5)	
CCRT	8 (40.0)	13 (65.0)	21 (52.5)	
RT Dose				*0.75*
≤66 Gy	12 (60.0)	10 (50.0)	22 (55.0)	
>66 Gy	8 (40.0)	10 (50.0)	18 (45.0)	
Total	20 (100)	20 (100)	40 (100)	

Abbreviations: **ECOG PS**, Eastern Cooperative Oncology Group Performance Status **Post-OP**, Post-Operative; **RT**, Radiotherapy; **CCRT**, Chemo-radiotherapy; **Gy**, Gray.

**Note 1**: All *P* values were estimated by using the Fisher's exact test.

**Note 2**: ECOG performance status was recorded by a radiation oncologist at the time of initial presentation at RT department.

**Note 3**: Factors that marked with “*” were used for match-paired.

**Table 2:** Association between promoter methylation of *FHIT*and clinical-pathological features in 40 oral cancer patients

	Methylation (%)	*P*
**Patient**		
Age		
≤50 years	21.36 ± 21.29^1^ (16/40)^2^	
>50 years	25.98 ± 22.67 (24/40)	0.52
Gender		
Female	4.76 ± 3.09 (2/40)	
Male	25.16 ± 22.17 (38/40)	**<0.001^3^**
Smoking		
No	14.61 ± 14.29 (6/40)	
Yes	25.82 ± 22.81 (34/40)	0.16
Betel nut chewing		
No	12.23 ± 14.01 (7/40)	
Yes	26.67 ± 22.78 (33/40)	**0.047^3^**
**Tumor**		
Histology		
Grade 1-2	23.17 ± 20.95 (35/40)	
Grade 3-4	30.91 ± 30.79 (5/40)	0.61
pT status		
pT1-3	15.83 ± 12.15 (27/40)	
pT4	28.13 ± 22.58 (13/40)	0.084
pN status		
pN0	23.70 ± 20.95 (33/40)	
pN1-2	26.58 ± 24.21 (7/40)	0.83
PNI		
No	21.91 ± 19.97 (31/40)	
Yes	31.79 ± 28.13 (9/40)	0.35
LVSI		
No	12.60 ± 9.61 (33/40)	
Yes	26.59 ± 23.25 (7/40)	**0.017^3^**

Abbreviation: **PNI**, peri-neural infiltration; **LVSI**, lymph-vascular space invasion;

1 Methylation% (mean ± SD);

2 Number in parentheses represents number of cases;

3 Bold value indicates *P* < 0.05.

Multivariate analysis also confirmed that *FHIT* promoter hypermethylation was an independent risk factor for predicting poor locoregional control (HR, 11.55; 95% CI, 1.41 – 99.9; *P* = 0.043; Table 3) and poor overall survival (HR, 3.06; 95% CI, 1.15 – 8.14; *P* = 0.036; Table 4). As expected, in addition to *FHIT* promoter hypermethylation, advanced pathological stage and very close surgical margin were also identified as independent factors for predicting shortened overall survival (Table 4).

**Table 3:** Hazard ratio for locoregional recurrence according to predictive factors

	HR (95% CI); *P* value	
	Univariate	Multivariate
**Patient factors**		
Age (≤50 vs. >50 years)	2.92 (0.71 – 12.01); 0.14	NA
Gender (female vs. male)	4.61 (0.25 – 86.58); 0.31	NA
Smoking (no vs. yes)	0.72 (0.08 – 6.77); 0.77	NA
Betel nut chewing (no vs. yes)	3.99 (0.28 – 56.86); 0.31	NA
ECOG PS (0-1 vs. ≥2)	2.34 (0.40 – 13.69); 0.62	NA
**Tumor factors**		
Histology (G1-2 vs. G3-4)	1.49 (0.58 – 3.83); 0.24	NA
Depth (≤submucosa vs. ≥muscle)	1.69 (0.41 – 8.02); 0.52	NA
PNI (no vs. yes)	1.98 (0.71 – 5.94); 0.28	NA
LVSI (no vs. yes)	1.52 (0.51 – 5.38); 0.59	NA
ECS (no vs. yes)	7.86 (<0.01 – >100); 0.96	NA
p-Stage (non-IVA/B vs. IVA/B)	7.05 (1.14 – 43.5); 0.036*	5.25 (1.08 – 25.57); 0.047*
**Treatment factors**		
Surgical margin (>1 vs. ≤1 mm)	6.36 (1.48 – 27.33); 0.011**	4.36 (1.22 – 15.5); 0.023**
Post-OP therapy (RT vs. CCRT)	2.83 (0.88 – 9.31); 0.086*	3.58 (0.48 – 26.7); 0.11
RT dose (≤66 vs. >66 Gy)	3.08 (0.67 – 14.15); 0.27	NA
**Gene promoter methylation**		
*FHIT* (no vs. yes)	14.55 (1.67 – >100); 0.039**	11.55 (1.41 – 99.9); 0.043**

Abbreviation: **CI**, confidence interval; **PNI**, peri-neural infiltration; **LVSI**, lymph-vascular space invasion; **ECS**, extra-capsular spreading; **ECOG PS**, Eastern Cooperation Oncology Group performance status; ***FHIT***, fragile histidine triad; **HR**, hazard ratio; **95% CI**, 95% confidence interval; **, *P*<0.05; *,*P* <0.10 but ≥0.05.

**Note 1**: The first group in all predictive factors was used as the reference group for estimating hazard ratio. For example, ‘≤50 years’ was the reference group (value = 1) in the factor of ‘Age.’

**Note 2**: Factors with a univariate *P* value of <0.10 were used for further multivariate analysis.

**Table 4:** Hazard ratio for overall survival according to predictive factors

	HR (95% CI); *P* value	
	Univariate	Multivariate
**Patient factors**		
Age (≤50 vs. >50 years)	1.52 (0.40 – 5.73); 0.54	NA
Gender (female vs. male)	1.18 (0.06 – 25.54); 0.91	NA
Smoking (no vs. yes)	0.99 (0.09 – 11.21); 0.99	NA
Betel nut chewing (no vs. yes)	4.61 (0.29 – 70.95); 0.27	NA
ECOG PS (0-1 vs. ≥2)	2.86 (0.89 – 9.20); 0.083*	1.93 (0.48 – 71.32); 0.56
**Tumor factors**		
Histology (G1-2 vs. G3-4)	3.17 (0.44 – 23.09); 0.25	NA
Depth (≤submucosa vs. ≥muscle)	1.19 (0.38 – 3.83); 0.63	NA
PNI (no vs. yes)	1.28 (0.39 – 4.20); 0.48	NA
LVSI (no vs. yes)	1.72 (0.52 – 5.84); 0.46	NA
ECS (no vs. yes)	9.36 (<0.01 – >100); 0.92	NA
p-Stage (non-IVA/B vs. IVA/B)	10.25 (1.64 – 64.6); 0.009*	2.91 (1.44 – 5.98); 0.020*
**Treatment factors**		
Surgical margin (>1 vs. ≤1 mm)	5.84 (1.58 – 21.63); 0.017**	2.24 (1.27 – 1.76); 0.032*
Post-OP therapy (RT vs. CCRT)	2.48 (0.93 – 6.61); 0.061*	1.83 (0.62 – 5.41); 0.38
RT dose (≤66 vs. >66 Gy)	1.98 (0.45 – 8.70); 0.58	NA
**Gene promoter methylation**		
*FHIT* (no vs. yes)	6.12 (1.98 – 3.09); 0.028**	3.06 (1.15 – 8.14); 0.036**

Abbreviation: **CI**, confidence interval; **PNI**, peri-neural infiltration; **LVSI**, lymph-vascular space invasion; **ECS**, extra-capsular spreading; **ECOG PS**, Eastern Cooperation Oncology Group performance status; ***FHIT***, fragile histidine triad; **HR**, hazard ratio; **95% CI**, 95% confidence interval; **,*P* <0.05; *,*P* <0.10 but ≥0.05.

**Note 1**: The first group in all predictive factors was used as the reference group for estimating hazard ratio. For example, ‘≤50 years’ was the reference group (value = 1) in the factor of ‘Age.’

**Note 2**: Factors with a univariate *P* value of <0.10 were used for further multivariate analysis.

We further explored the outcome-predicting role of three independent factors (promoter methylation, stage, and very close surgical margin). Both Kaplan-Meier (Fig. S6) and Cox regression analysis (Table S4) demonstrated that patients with any two of these three factors or all three factors had poorer clinical outcomes than those with only one or no risk factor.

### Epigenetic interventions enhance radiosensitivity, *in vivo*

**Figure 8:**
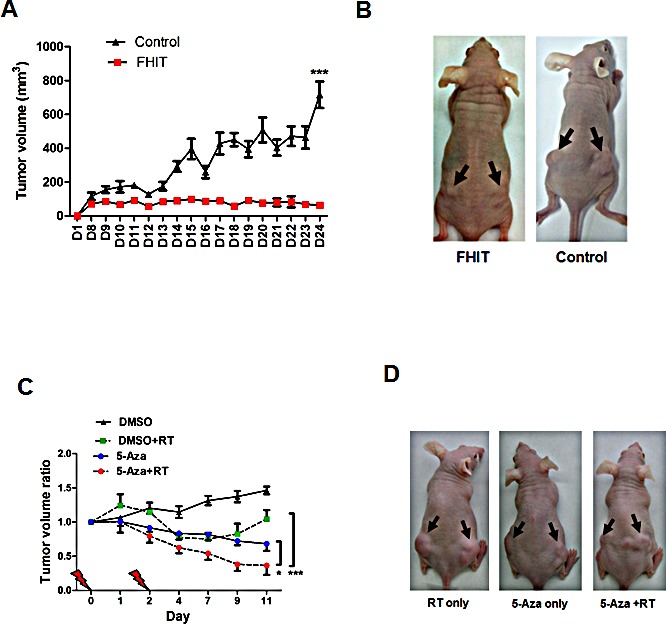
Effect of *FHIT* expression and epigenetic intervention on tumor growth in an animal model (A) The effect of *FHIT* on tumor growth *in vivo* was also determined by a nude mice model. OML1-R cells stably transfected with *FHIT* or empty vector (control) were injected subcutaneously into athymic nude mice. Tumor volumes were measured daily. (B) Representative examples of tumors formed in nude mice following injection of OML1-R cells overexpressing *FHIT* (left panel, arrow) and empty plasmid control (right panel, arrow). Data are expressed as means ± SD (n=4). (C) The effect of irradiation (RT), DNA methylation inhibitor (5-Aza) and combined treatment on radiosensitivity of OML1-R cells *in vivo*. Radioresistant OML1-R oral cancer cells were injected subcutaneously into athymic nude mice. Tumors were allowed to grow to > 500mm^3^ and were then treated with two fractions of 4-Gy irradiation, 5-Aza, or both at the indicated time points (Days 0 and 2). Tumor volumes were measured daily and expressed as tumor volume ratios (tumor volume on the measured day / initial tumor volume on day 0). Statistical significances were observed when comparing tumor volume ratios between 5-Aza + RT and 5-Aza alone (*, *P* < 0.05) or between 5-Aza + RT and RT alone (***, *P* < 0.001). (D) Representative examples of OML1-R tumors formed in nude mice treated with RT only (left panel, arrow) or 5Aza + RT on day 11 (right panel, arrow).

Next, we tested the role of *FHIT* in tumor growth and radiosensitivity in an *in vivo* xenograft mouse model. Compared to empty vector control, *FHIT*-overexpressing OML1-R cells exhibited significantly less tumor growth than mice injected with mock-transfected OML1-R cells (Fig. 8A,B), thus confirming high tumor suppression by *FHIT, in vivo*.

Since *FHIT* was epigenetically silenced in OML1-R cells, and radioresistance correlated with aberrant epigenetic changes *in vitro*, we examined the possibility of *in vivo* epigenetic interventions to resensitize OML1-R cells to irradiation. Mice were subcutaneously injected with OML1-R cells, followed by treatment of radiotherapy (RT, 4-Gy by 2 fractions), i.p administration of 5-Aza or concurrent 5-Aza and RT. Significant reduction in tumor volumes was observed in mice treated with combined 5-Aza and RT when compared with 5-Aza or RT alone (Fig. 8C,D) demonstrating a synergistic effect and potential treatment direction of combining demethylating agents with RT.

## DISCUSSION

In the therapy of oral cancer, after massive irradiation, it is believed that radioresistant cancer cells harbored in the surgical bed are the likely sources of locoregional recurrence [27]. Moreover, post-irradiation epigenetic changes have been suggested to play a significant role in eliciting radioresistance of irradiated cancer cells [18, 28-29]. These phenomena make “epimutations” attractive bio-targets for both predicting clinical outcomes and enhancing treatment efficacy. However, the role of epigenetic changes in radioresistant oral cancer has not been fully explored.

In the present study, we used a microarray-based strategy to identify genes differentially methylated in an *in vitro* model of acquired oral cancer radioresistance. One particularly interesting microarray target, *FHIT* (fragile histidine triad), was found epigenetically silenced in radioresistant cells. Moreover, restoring expression of epigenetically repressed *FHIT* resensitized radioresistant oral cancer cells to ionizing radiation. Remarkably, epigenetic interventions using DNMT inhibitors significantly enhanced the radiosensitivity of resistant oral cancer cells, *in vivo.* Our results together with previous observations [30-31] suggest that concomitant treatment of DNA-demethylating agents and radiotherapy is a new treatment direction for this aggressive malignancy.

*FHIT* has been reported to be involved in several tumor-suppressive processes, such as enhancing apoptosis [32] and inhibiting tumor growth [33]. On the other hand, reduced *FHIT* expression has been correlated with increased genome instability [34], decreased DNA damage-induced cell killing [35], and solid tumor progression [36]. However, the role of *FHIT* in the radioresistance of human cancers is less explored. Herein, we observed a role of *FHIT* in initiating radioresistance in irradiated oral cancer cells. *In vivo* and human sample studies validated both therapeutic and predictive efficacy, warranting further clinical trials to confirm these clinical roles.

Ionizing radiation has been reported to induce ATM-Chk2-dependent checkpoint signaling followed by a G2/M cell cycle arrest [37-39], believed to drive sub-lethally damaged cancer cells toward apoptosis [40-42]. As a result, loss of pChk2 may facilitate cancer cells' escape from checkpoint-dependent apoptosis, leading to a high level of radioresistance [43-44]. Restoring *FHIT* expression has been observed to restore pChk2 activity and then re-radiosensitization of oral cancer cells [45]. Our data confirmed this observation and re-emphasized the role of reduced apoptosis in the development of radioresistance.

Epigenetic silencing of *FHIT* has been observed in several human cancers [46-49]. In this study, high promoter methylation of *FHIT* (>10%) can be observed in 55% (22/40) of oral cancer patient samples. Recently, Mielcarek-Kuchta *et al.* demonstrated that, by using methylation specific PCR (MSP), promoter methylation of *FHIT* was only observed in 1.8% of 53 cases of oral and oropharyngeal cancer [50]. This discrepancy may be due to a technical difference such that a quantitative bisulphite pyrosequencing was applied in our study. It is also interesting to point out that there is a group of patients with a relatively low methylation of *FHIT* (<10%) but also negative staining of FHIT (Fig 7B). Other mechanisms such as genetic alteration may play a role in the down-regulation of FHIT in this group of oral cancer patients [51-53].

Nevertheless, the present study is the first to demonstrate that epigenetic silencing of *FHIT* can initiate radioresistance in irradiated human oral cancers cells. *FHIT* is located in the fragile site of chromosome 3p [19], likely a frequent site of DNA damage following irradiation. As a result, repressive histone marks and DNA methylation eventually accumulate within this region, as previously observed [54-55]. Thus, we may conclude that through recruitment of polycomb repressors (*e.g.*, EZH2) and DNMTs, repeated radiation-induced DNA damages results in epigenetic silencing of *FHIT*, likely initiating post-irradiation radioresistance.

Epigenetic therapy has been recognized as an emerging approach for treating numerous solid cancers [56], particularly in combination with radio- or chemotherapy [29, 57-60]. For examples, decitabine (5-Aza) has been reported to enhance radiosensitivity through activating G2/M checkpoint responses, inducing apoptosis in osteosarcoma [61], breast cancer [62], colorectal cancer [63], medulloblastoma [64], and head-and-neck cancers [30-31]. Our results may provide an explanation for the phenomenon that inhibitors of DNMT or EZH2 reverse repressive epigenomes to resensitize radioresistant cancer cells to ionizing radiation.

A more systematic assessment of radiation enhancement by epigenetic interventions has been conducted in head-and-neck cancer cells [31]. However, the role of promoter hypermethylation of *FHIT* may be underestimated. Based on our data, promoter hypermethylation of *FHIT* demonstrated dual roles not only in predicting clinical outcomes, but also implicating a treatment target. Thus, further clinical trials to investigate a combined effect of radiotherapy and epigenetic interventions are justified, especially in targeting restoration of *FHIT* [65].

Unexpected close surgical margin after a radical surgery is frequently encountered in resected oral cancer patients [66-67]. In addition to surgeon effects, unexpected close surgical margins have been recognized partially as an aggressive cancer behavior [68-69]. That is, bio-aggressive tumors harboring highly migratory cancer cells demonstrate a higher incidence of unexpected close surgical margins than that of bio-indolent tumors [16]. Clinical data supported this biological reasoning. Patients with close surgical margins demonstrate a higher risk of cancer recurrence and poorer survival than patients with free surgical margin [67, 70-72]. However, close margin alone does not self-sufficiently guide post-operative adjuvants [1, 66, 73-74], this clinical discrepancy reveals a possibly mixed population in these patients. Therefore, exploring a predictive biomarker is valuable for further patient stratification. We have previously confirmed this point of view by identifying promoter hypermethylation of *DAPK* as a predictive factor for locoregional control [16], and here, we further explored another potential prognostic marker, *i,e.,* promoter hypermethylation of *FHIT*, as a useful predictor of both locoregional control and overall survival, in irradiated oral cancer patients.

In conclusion, we have identified *FHIT* as epigenetically silenced by DNA methylation and histone modification in radioresistant oral cancer cells. Epigenetically or ectopically restoring *FHIT* suppresses tumor growth and enhances radiosensitivity in oral cancer cells. In conjunction with pathological factors, *FHIT* promoter hypermethylation demonstrates a promising role in predicting clinical outcomes, stratifying high-risk patients, and implicating new potential treatment targets. The radiosensitizing effect of DNMT and/or EZH2 inhibitors warrants further investigation in randomized clinical trials.

## MATERIALS AND METHODS

### Ethical considerations

All experiments involving human samples were conducted in accordance with the Helsinki Declaration of 1975, as revised in 2000. This study was also approved by the Institution Review Board (IRB) of the Buddhist Dalin Tzu Chi Hospital, Chia-Yi, Taiwan (approval number: B09804009). All animal experiments were approved by the Animal Experimentation Ethics Committee of the National Chung Cheng University, Taiwan.

### Patient samples

Between Aug 2004 and Dec 2008, we retrospectively selected 40 match-paired oral cancer patient samples based on the distance of surgical margin (Table 1 and Supplementary Fig. S1). Pathological features were prospectively defined at the time of radical surgery by using a checklist, and all of pathological reports were confirmed by two independent pathologists according to the requirement of Taiwan Medical Accreditation on Cancer Center [16]. A cancer case manager and a radiation oncologist independently reviewed all patient records and data discrepancies were resolved by consensus. Locoregional control and overall survival were defined as study end points, as previously described [67, 75]. Cancer staging was defined according to the American Joint Committee on Cancer, the 6^th^ edition [76]. For DNA extraction, paraffin-embedded tissue blocks were re-sectioned according to our IRB protocol. Tumor-burden areas that contained >70% cancer cells were microdissected under microscopy.

### Clinical treatment modality

Radical surgery with a curative intent was conducted in all 40 patients. In primary operation, 19 patients had underwent bone resection, either partial mandibulectomy (*n* = 16) or maxillectomy (*n* = 3). In neck management, supra-omohyoid (*n* = 25) or modified radical neck dissection (*n* = 15) was conducted. All 40 patients were treated with post-operative RT with (n=21) or without (n=19) cisplatin-based chemotherapy, as previously reported [67, 75].

### Cell culture and epigenetic treatment

Oral cancer cell lines OML1, OCSL, SAS (obtained from Dr. Yong-Kie Wong, Department of Dentistry, Taichung Veterans General Hospital, Taichung, Taiwan) were propagated with RPMI Medium 1640 (Gibco, Grand Island, NY) containing 10% fetal bovine serum (FBS) (Invitrogen, Carlsbad, CA) and 50 units/ml of penicillin/streptomycin (Invitrogen). The SCC4 and SCC25 cell lines were cultured in a 1:1 mixture of DMEM/F12 (Gibco) supplemented with 10% fetal bovine serum (FBS, Invitrogen) and 50 units/ml of penicillin/streptomycin (Invitrogen). Primary human oral keratinocytes (HOK, ScienCell, Carlsbad, CA) which were isolated from human oral mucosa were cultured in oral keratinocyte medium (OKM, ScienCell). For epigenetic treatment, 2 × 10^5^ cells were seeded onto a 90-mm plate and treated with 0.5μM of 5′-aza-2′-deoxycytidine (5-Aza; Sigma, St. Louis, MO) for 72 hours; 0.5μM Trichostatin A (TSA, Sigma) for 12 hours; or 10μM of EZH2 inhibitor, GSK343 (Cayman, Ann Arbor, MI) for 72 hours. For combination treatment, cells were either treated with 72 hours of 5-Aza followed by 12 hours of TSA or 72 hours of 5-Aza followed by 72 hours of GSK343. Medium was changed and new drug was added every 24 hours.

### Establishment of a radioresistant subline by using fractionated irradiations

For studying the role of epigenetics in oral cancer radioresistance, we established a radioresistant subline of the oral cancer cell line OML1, using hypo-fractionated irradiations (see schema in Fig. 1A). Briefly, one day before irradiation, 1 × 10^6^ of the parental (“OML1-P”) cells were seeded into 10-cm cell culture plates. A fraction of 5-Gy irradiation was then delivered by a 6-MV linear accelerator (Elekta, Sweden). For achieving equivalent dose distributions, two 1.5-cm biomaterial-equivalent boluses were applied on both up- and down-side of the culture plate (*i.e.*, arranged like a sandwich). After irradiation, cells were allowed to recover for 5-7 days. These procedures were then repeated for 10 sessions, up to a total dose of 50 Gy, to obtain the radioresistant cell subline OML1-R.

### Microarray-based methylation analysis

Bisulphite-modified DNA was subjected to methylation analysis using an Illumina Infinium Human Methylation27 microarray (Illumina, San Diego, CA). Duplicate experiments were then performed to determine methylation β-values of specific probes, defined as the ratio of the methylated signal intensity to the sum of methylated and unmethylated signal of a probe. A β-value of 0 represented un-methylation while 1.0 represented full methylation. For selection of differentially methylated probes, the following criteria were applied: 1) probes were present in “CpG islands”; 2) probes having a mean β-value of <0.5 (for hypermethylated probes) or >0.5 (for hypomethylated probes) in the control groups (OML1-P cells, as compared to the radioresistant daughter cells); and 3) when comparing mean β-values between experimental groups, probes with both *P* <0.05 and differential changes of β-value ≥0.2 were obtained.

### *In vivo* tumorigenicity assay

Four 3-week-old athymic nude mice (BALB/cAnN.Cg-Foxn1nu/CrlNarl) were obtained from National Laboratory Animal Center, Taiwan. All mice were kept under specific pathogen-free conditions using laminar airflow racks, with free access to sterilized food and autoclaved water. All experiments were performed under license from the Animal Experimentation Ethics Committee of the National Chung Cheng University. 1 × 10^6^ OML1-R cells stably transfected with empty vector (control) or pcDNA3.1/*FHIT* were re-suspended in 0.1ml 1:1 PBS/ Matrigel (BD Bioscience, San Jose, CA) mixture. The cell suspension was then injected subcutaneously into the flank of each mouse (day 0). Tumor length (L) and width (W) were measured daily with calipers, and tumor volume calculated using the formula (L × W^2^/2). At the end of each experiment, all mice were sacrificed. To test the radiosensitizing effect of 5-Aza, 1 × 10^6^ OML1-R cells were injected subcutaneously into the flank of each mouse. When the tumor volume exceeded 500mm^3^, two fractions of 4-Gy irradiation with 5-Aza (at a dose of 0.5mg/kg dissolved in 0.01M DMSO) or vehicle only (DMSO) were delivered IP, as shown in Fig. 6C. Tumor volume was monitored and presented as the tumor volume growth ratios (final volume / initial volume).

### Statistical analysis and definitions

SPSS (version 12.0; SPSS Inc., Chicago, IL, USA) was used for statistical analyses, as follows. Kaplan-Meier analysis was used to estimate survival and cancer control rates; the log-rank test was applied to assess curve differences between groups; and Cox proportional hazard regression was used to perform univariate and multivariate analyses for time-to-event endpoints. All time-to-event analyses calculated the time interval from the day of pathological diagnosis to the day of corresponding end events, as previously described [67, 75]. A DNA methylation level at 10%, which is the average methylation level in OML1-P cells (Fig. 2C), was used as a cut-off. Independent variables that achieved a statistical significance (P < 0.05) or a statistical trend (*P* < 0.1 but ≥ 0.05) in univariate analysis were used in multivariate analysis. Comparison of grouped quantitative data was conducted by using student t test. For demarcating the effective size, 95% confidence (95% CI) intervals were provided in conjunction with point-estimated hazard ratios (HRs), in addition to a conventional *P* value. All tests were two-tailed and considered to be statistically significant when *P* <0.05.

Additional Methods can be found in supplementary information.

## SUPPLEMENTARY MATERIAL, FIGURES AND TABLES


