# Supplementary material for: DNA methylome analysis identifies epigenetic silencing of FHIT as a determining factor for radiosensitivity in oral cancer: an outcome-predicting and treatment-implicating study
Source: Oncotarget. 2014 Nov 25;6(2):915–34. doi: 10.18632/oncotarget.2821 (PMC4359265; doi:10.18632/oncotarget.2821)
Supplement: Supplementary file 1 [file oncotarget-06-915-s001.pdf]

DNA methylome analysis identifies epigenetic silencing of FHIT as a determining factor for radiosensitivity in oral cancer: an outcome-predicting and treatment-implicating study

Supplementary Material

RNA extraction and quantitative reverse transcription-PCR

Total RNA from cell lines was extracted by using Trizol (Invitrogen), as previously described.[1] In brief, 1μg of total RNA was treated with DNase I (amplification grade, Invitrogen) before first-strand cDNA synthesis by using reverse transcriptase (Superscript II RT, Invitrogen). PCR reactions were carried out using ABI StepOne real-time PCR system (Applied Biosystems, Foster city, CA). Specific primers were used accordingly (Table S1). Relative expression of *FHIT* was calculated by using the comparative Ct method.

Analysis of cell survival after radiation stress by using colony formation assay

1 x 10^3 cells were seeded on a 6-cm plate on the day before experiment. Cells were irradiated with 10 Gy and cultured for several days. For quantitation of cell numbers, cells stained with 0.4% crystal violet (Sigma) were counted at OD580 by using GeneQuant 1300 spectrophotometer (GE healthcare, UK).

Genomic DNA extraction and bisulfite conversion

As described previously[1], genomic DNA was extracted and then bisulphite modified (*i.e.*, nonmethylated cytosines deamined to uracil) by using Geneaid Kit (ID, GT100, Taiwan) and EZ DNA Methylation Kit (Zymo Research, Orange, CA), respectively.

Bisulfite pyrosequencing

Bisulfite-modified DNA was amplified using PCR. A tailed reverse primer in combination with a biotin-labeled universal primer was used. PCR was amplified in a 25 μl reaction, which contained 12.5μl

of 2x RBC Sensizyme Hotstart Taq premix (RBC Bioscience, Taiwan), 0.5μM of each primer, 1μl DMSO, and 4 μl of bisulfite-modified DNA. PCR program was as follows: 95°C for 5min; 50 cycles of 95°C for 30 seconds, 56°C for 1 minute, 72°C for 45 seconds; and, a final extension at 72°C for 7 minutes. A total of 1.5 μl of each PCR products was analyzed on a 2% agarose gel before pyrosequencing. Pyrosequencing was conducted using PyroMark Q24 (Qiagen). Pyro Gold Reagents (Qiagen) was applied according to the manufacturer's protocol.

Methylated-DNA capture PCR (MBDCap-PCR)

Enrichment of methylated DNA was performed using the Methylminer kit (Invitrogen), as described previously [2]. Briefly, 1μg of sonicated DNA was incubated at room temperature on a rotator mixer in a solution contained 3.5μg of MBD-Biotin Protein coupled to M-280 Streptavidin Dynabeads. Non-captured DNA was removed by collecting beads on a magnet and washing three times with Bind/Wash Buffer. Enriched, methylated DNA was eluted from the bead complex with 1 M NaCl and purified by ethanol precipitation. Amount of methylated DNA was measured by using quantitative-PCR according to specific primers (Table S1). A standard curve generated by input DNA against 200 pg of captured DNA was delineated.

Quantitative ChIP-PCR

A total of 1×10^6 cells were cross-linked with 1% fresh formaldehyde and then washed with cold 1 x PBS in the presence of protease inhibitor. Cell were homogenized and their chromatin was subjected to chromatin immune-precipitation (ChIP) pull down by magnetic Dynal beads (Invitrogen) and antibodies against H3K27me3 (Merck Millipore, Darmstadt, Germany). Amount of ChIP DNA was measured by using quantitative-PCR analysis. A total of 200 pg of pull-down DNA were amplified using *FHIT* promoter specific primers (Table S1) and then were quantified as the percentage of input DNA. The

qPCR reactions were carried out by using ABI StepOne Real-Time PCR systems (Applied Biosystems, Foster city, CA).

Immunohistochemical analysis of *FHIT*

Paraffin-embedded oral cancer tissues of the above-mentioned patient samples were retrieved from the department of Pathology at the Dalin Tzu Chi Hospital after an approval of IRB. Tissue sections were de-waxed in xylene and re-hydrated in alcohol. Antigen retrieval was performed by heating each section at 100°C for 20 min in 10mM sodium citrate buffer (pH 6.0). The immunohistochemistry (IHC) procedure followed a standard protocol by using an anti-FHIT polyclonal antibody (1:100, abcam, Cambridge, MA) and Novolink^{TM} Min Polymer Detection System (Leica, Wetzlar, Germany). IHC result was assessed by 2 pathologists independently. Discrepancy was resolved by consensus. A ≤10% of cancer cells that were stained was defined as negative FHIT expression.

Plasmid constructing and transfection

The complete coding sequence (CDS) of *FHIT* was amplified using PCR with specific primers (Table S1). The amplified PCR products were then cloned into pcDNA3.1/myc-His mammalian expression vector. *FHIT* expression vector or empty vector was transfected into designated cells using TransIT-LT1 Transfection Reagent (Mirus, Madison, WI) according to the manufacturer's protocol. Cells were then selected for stable clones using medium containing 400μg/ml Geneticin (G418, Invitrogen).

Knocking down *FHIT* by using shRNA

The shRNA targeted to *FHIT* was acquired from the National RNAi Core Facility Platform at the Institute of Molecular Biology/Genomic Research Center, Academia Sinica, Taiwan. Briefly, to prepare the shFHIT and shEZH2 lentivirus, 293T cells were transfected with shRNA (TRCN0000051173 and TRCN0000303423), pMDG, and pCMVR 8.91 using ProFection Mammalian Transfection System

(Promega). Infected cells were selected by incubating with puromycin (2 μg/mL; Sigma) for at least 2 days.

Protein extraction and Western blot

Cells were lysed with 100μl of PRO-PREP Protein Extraction Solution (iNtRON Biotechnology, Korea) according to the manufacturer's protocol. Samples and pre-stained marker were loaded to a 12.5% polyacrylamide gel for electrophoresis. The proteins were then transferred onto a PVDF membrane by using the Mini Trans-Blot Electrophoretic Transfer Cell system (Bio-Rad, Hercules, CA) at 400 mA for about 90 minutes. After the transfer, the membrane was incubated with 5% non-fat milk in 1X TBST for 1 hour at room temperature. Then primary antibodies, rabbit anti-p53 (1:1000, Cell Signaling, Beverly, MA), rabbit anti-Chk2 (1:1000, Cell Signaling), rabbit anti-pChk2 (Thr68) (1:1000, Cell Signaling), rabbit anti-p21 (1:1000, Cell Signaling), rabbit anti-EZH2 (1:1000, Cell signaling), mouse anti-Flag (1:1000, Santa Cruz), or mouse anti-GAPDH antibody (1:2000 Thermo) was diluted with 5% non-fat milk in 1X TBST and was added into the membrane followed by incubation for overnight at 4°C. The membrane was then washed by using 1X TBST at room temperature for 3 times. The secondary antibody conjugated with horseradish peroxidase (anti-mouse, 1:4000 or anti-rabbit 1:4000, Thermo Scientific, Rockford, IL) was also diluted with 5% non-fat milk in 1X TBST and incubated with membrane at room temperature for 1 hour. The membrane was washed with 1X TBST at room temperature for 3 times. The Immobilon Western Chemiluminescent HRP Substrate (Merck Millipore) was prepared by mixing equal volumes of the HRP Substrate Luminol Reagent as well as the HRP Substrate Peroxide Solution and added onto the membrane. Finally, the chemiluminecent was detected by using BioSpectrum 2D Imaging System (UVP, Upland, CA).

Flow cytometry analysis for cell cycle and apoptosis

Cell cycle was analyzed by flow cytometry, as described previously [1]. In brief, after trypsinization and fixation, cells were treated with 0.1mg/ml RNase, followed by staining with 10 μg/ml

propidium iodide (PI; Sigma). The percentage of cells at different phase of cell cycle was analyzed by using FACScan flow cytometer (Becton Dickinson, Franklin Lakes, NJ). The software cell quest (Becton Dickinson) was applied. The cell cycle distribution was quantified by using the software ModFit LT (Becton Dickinson). For apoptosis analysis, cells were treated with Annexin V-FITC and PI using apoptosis detection kit (Strong Biotech Corporation, Taiwan). Anexin V-FITC and PI binding were analyzed by flow cytometry.

References

1. Chou JL, Su HY, Chen LY, Liao YP, Hartman-Frey C, Lai YH, Yang HW, Deatherage DE, Kuo CT, Huang YW, Yan PS, Hsiao SH, Tai CK, Lin HJ, Davuluri RV, Chao TK, et al. Promoter hypermethylation of FBXO32, a novel TGF-beta/SMAD4 target gene and tumor suppressor, is associated with poor prognosis in human ovarian cancer. Lab Invest. 2010; 90(3):414-425.
2. Rodriguez BA, Frankhouser D, Murphy M, Trimarchi M, Tam HH, Curfman J, Huang R, Chan MW, Lai HC, Parikh D, Ball B, Schwind S, Blum W, Marcucci G, Yan P and Bundschuh R. Methods for high-throughput MethylCap-Seq data analysis. BMC Genomics. 2012; 13 Suppl 6:S14.

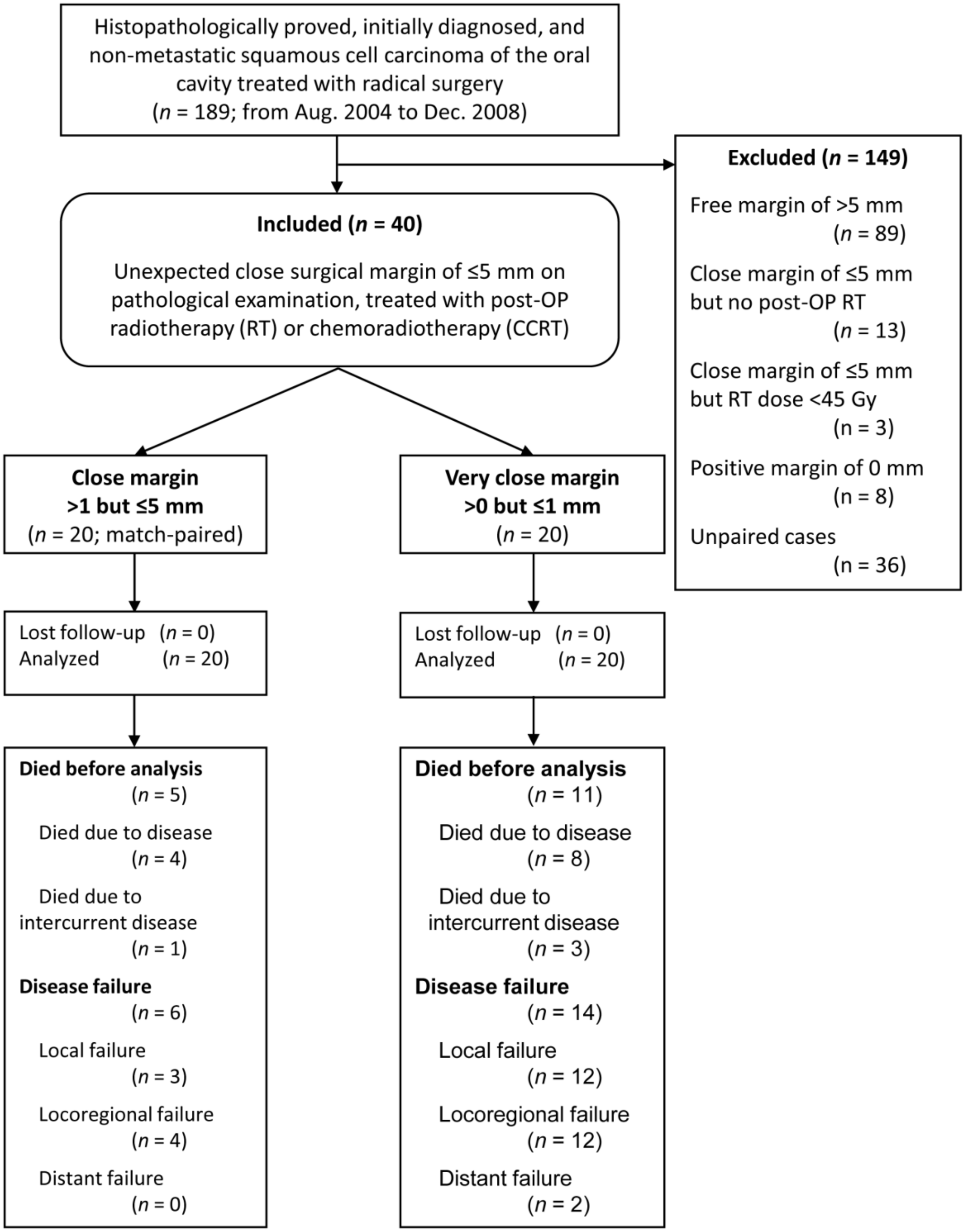

Figure. S1. Patient allocated flow chart according to the CONSORT statement.
Abbreviation: **Post-OP**, post-operative; **RT**, radiotherapy; and, **CCRT**, chemoradiotherapy.

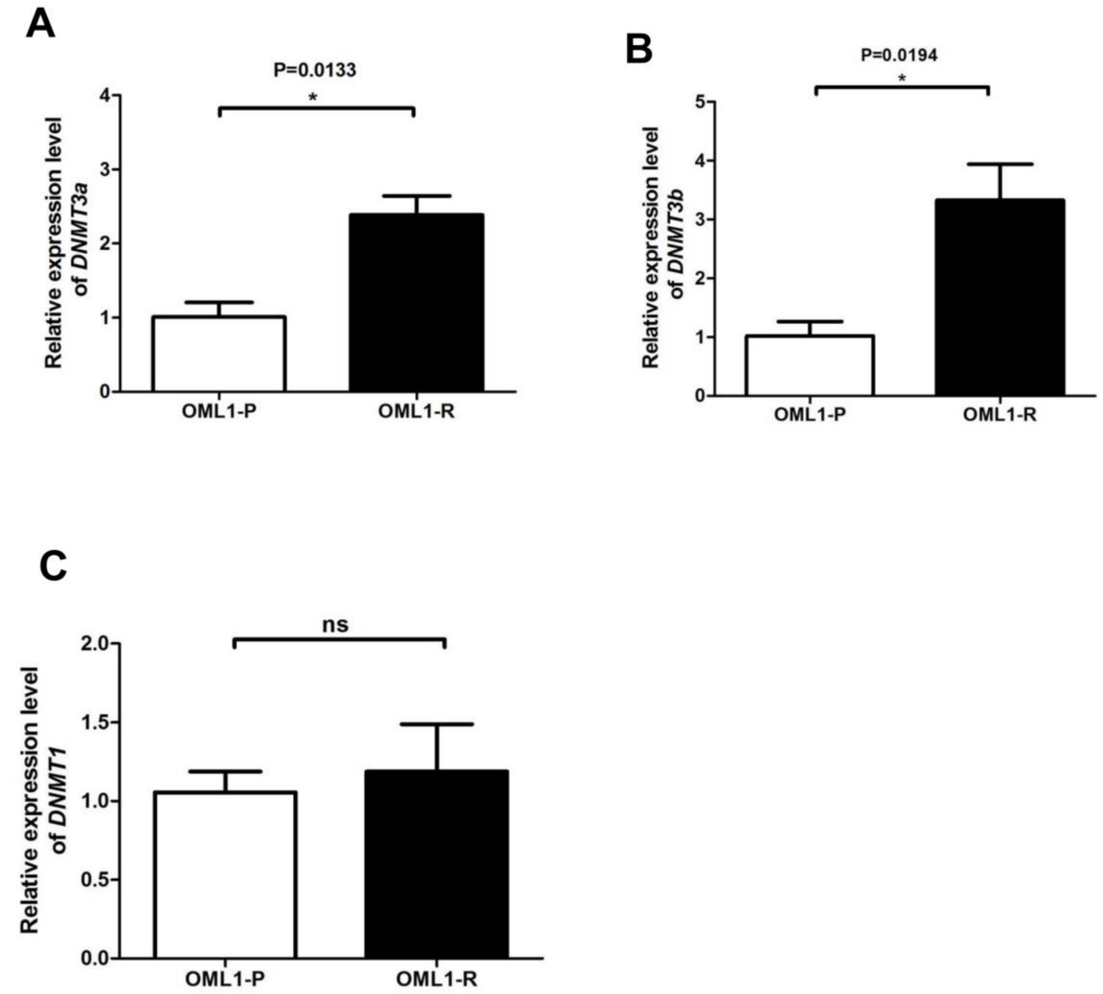

Figure S2. Expression of (A) DNMT3a; (B) DNMT3b; and (C) DNMT1 in OML1-P and OML1-R cells. mRNA expression was determined by qRT-PCR. Data in histograms are expressed as means $\pm$ SD (n=3).

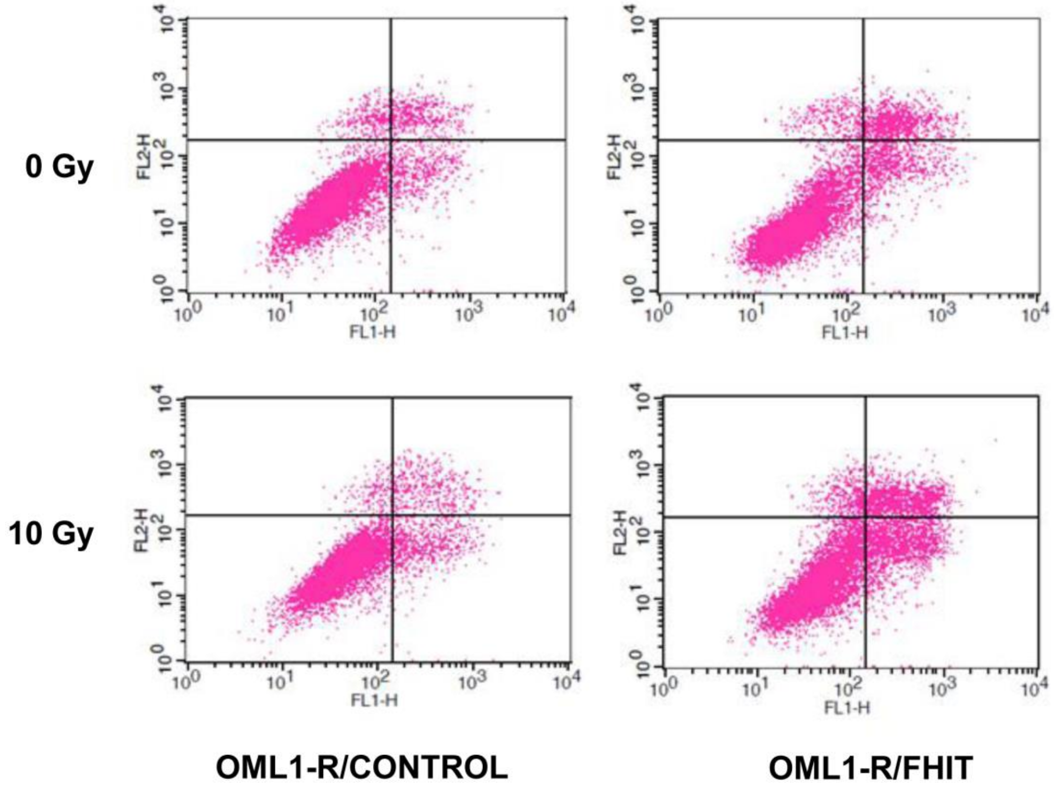

Figure S3. Representative flow cytometry result of Annexin V/PI apoptosis assay in control or *FHIT*-overexpressed OML1-R cells irradiated by 0 or 10-Gy irradiation.

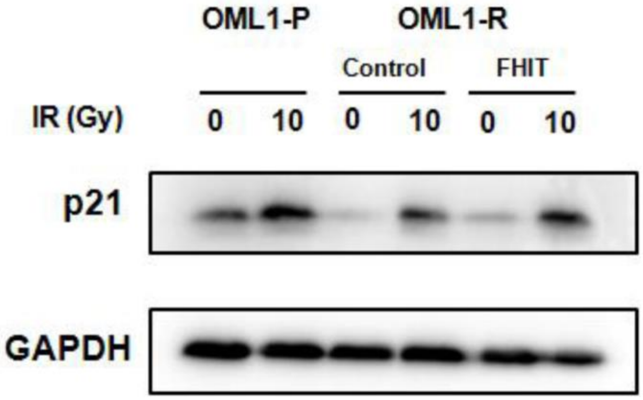

Figure S4. Western blotting analysis of p21 and GAPDH (loading control) in OML1-P and *FHIT*-overexpressing OML1-R cells. Overexpression of *FHIT* partially restored p21 expression in OML1-R cells irradiated with 10-Gy irradiation.

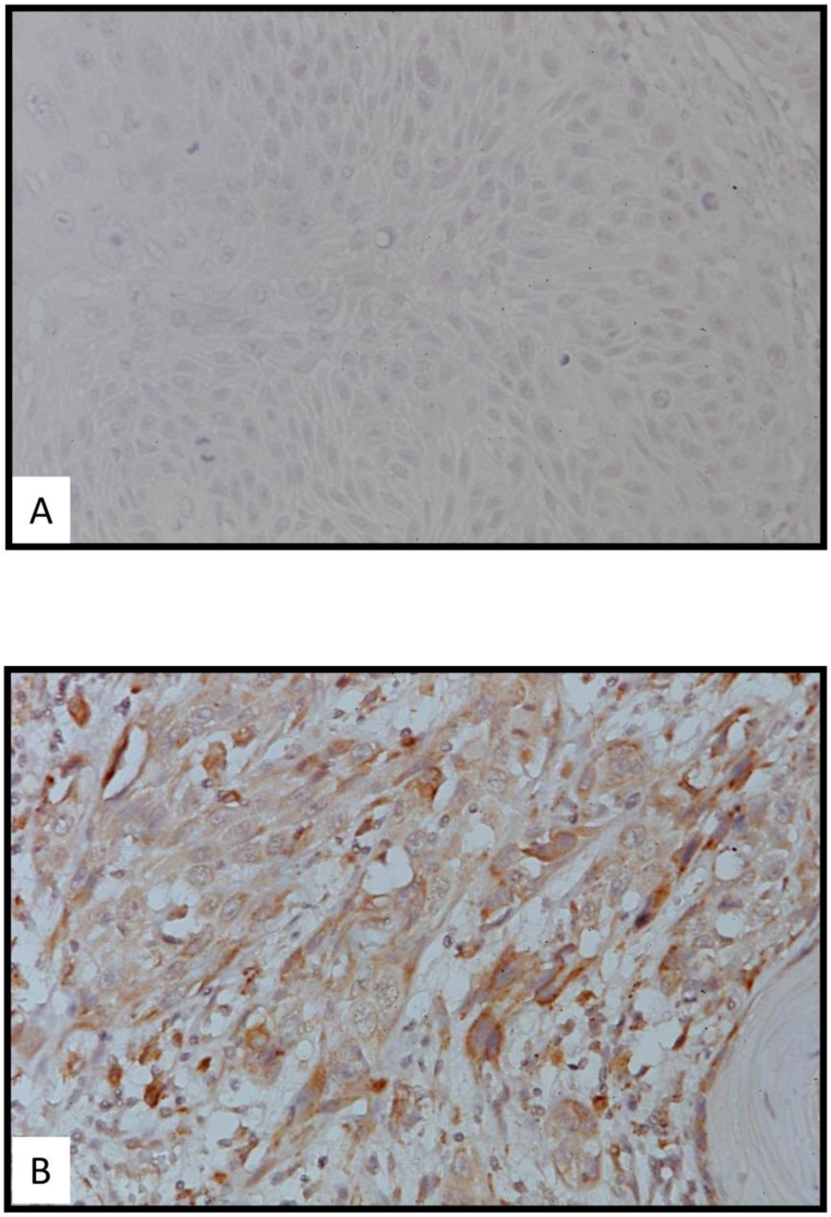

Figure S5. Representative images of immunohistochemistry (IHC) examination of FHIT in oral cancer patient samples. Samples with (A) low and (B) high expression of FHIT are shown (x400).

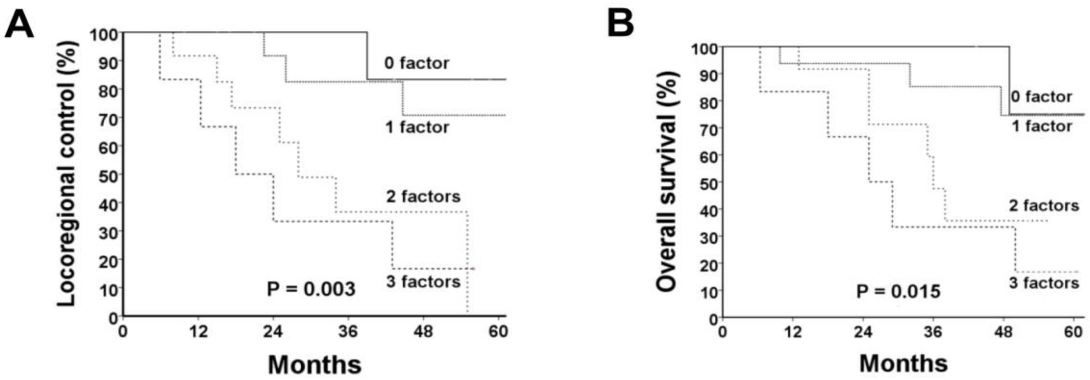

Figure S6. Kaplan-Meier analysis demonstrated that patients with any two or three risk factors (very close surgical margin, advanced pathological stage, and *FHIT* promoter hypermethylation) demonstrated lower locoregional control rates (**A**, *P* = 0.003) and shorter overall survival (**B**, *P* = 0.015) than those of patients with only one or no risk factor.

Table S1: Primers used in this study

**Bisulphite Pyrosequencing**	
FHIT_Pyro_F	TGAGGGATTAGGGAATAGAGGGTAAA
FHIT_Pyro_R-UNIVR*	agctggacatcacctcccacaacgCCCCCCCCTAAATCAAAATT ACTATCAC
Biotinylated-UNIVR	agctggacatcacctcccacaacg
FHIT_Pyro_Seq	GGGATTGTAATTTTTAGAAGA
**RT-PCR**	
FHIT_RT_F	GGCTGGAGACTTTCACAGGAAT
FHIT_RT_R	GCTGCCATTTCCTCCTCTGA
EZH2_RT_F	CTTGTGACAGTTCGTGCCCTT
EZH2_RT_R	TGTGTGTTGCACTGTGCTTTGC
Dnmt1_RT_F	CCCCTGAGCCCTACCGAAT
Dnmt1_RT_R	CTCGCTGGAGTGGACTTGTG
Dnmt3a_RT_F	GCCGAATTGTGTCTTGGTGGATGACA
Dnmt3a_RT_R	CCTGGTGGAATGCACTGCAGAAGGA
Dnmt3b_RT_F	CATTGCTGTTGGAACCGTGAA
Dnmt3b_RT_R	TCCGCCAATCACCAAGTCAA
GAPDH_RT_F	CCCCTTCATTGACCTCAACTACAT
GAPDH_RT_R	CGCTCCTGGAAGATGGTGA
**ChIP-PCR**	
FHIT _ChIP PCR_F	TTGTCTTCTGTGGTCAGTGTTTCC
FHIT _ChIP PCR_R	CCAAGATGGCCGCTTGTCT
GAPDH_ChIP PCR_	TTGACTCCCTAGTGTCCTGCTG
GAPDH_ChIP PCR_	CCTACTTTCTCCCCGCTTTTTT
**MBDcap-PCR**	
FHIT_MBDcap PCR_F	TTGTCTTCTGTGGTCAGTGTTTCC
FHIT_MBDcap PCR_R	CCAAGATGGCCGCTTGTCT
**Plasmid construction**	
FHIT_cDNA_BamH I_F	GAGGATCCGACATGTCGTTCAGATTTGGCC
FHIT_cDNA_EcoRI_R	GAGAATTCCTGAAAGTAGACCCGCAGAGCTG

Table S3: Gene Ontology (GO) biological process analysis of most significant hypermethylated genes in OML1-R cells using DAVID Bioinformatics Resources 6.7

Term	Count	P-values
GO:0002381~immunoglobulin production during immune response	3	5.71E-03
GO:0051640~organelle localization	5	9.03E-03
GO:0009100~glycoprotein metabolic process	7	9.21E-03
GO:0010605~negative regulation of macromolecule metabolic process	14	1.32E-02
GO:0043062~extracellular structure organization	6	1.50E-02
GO:0016051~carbohydrate biosynthetic process	5	1.51E-02
GO:0031123~RNA 3'-end processing	3	2.37E-02
GO:0042278~purine nucleoside metabolic process	3	2.71E-02
GO:0045017~glycerolipid biosynthetic process	4	3.41E-02
GO:0006259~DNA metabolic process	10	3.60E-02
GO:0045934~negative regulation of nucleobase, nucleoside, nucleotide and nucleic acid metabolic process	10	3.84E-02
GO:0051648~vesicle localization	3	4.25E-02
GO:0032465~regulation of cytokinesis	2	4.36E-02

Table S4: Estimated risks of post-irradiation clinical outcomes according to combination of very close surgical margin of ≤1 mm, pathological stage IVA-B, and *FHIT* promoter hypermethylation

	HR (95% CI)		
	Any 2 or all 3 factors	Any 1 or no factor	*P*
Locoregional recurrence	15.51 (3.16 – 76.97)	1	**0.001****
Local recurrence	12.84 (2.39 – 68.98)	1	**<0.001****
Disease failure at any site	14.89 (3.13 – 70.87)	1	**0.002****
Death from any cause	7.59 (1.88 – 30.89)	1	**0.003****

Abbreviations: ***FHIT***, fragile histidine triad; **HR**, hazard ratio; **95% CI**, 95% confidence interval.

Note 1: All hazard ratios were estimated by using Cox proportional hazard regression (**, *P* <0.05).

Note 2: Patients with no any factor of very close margin (≤1 mm), *FHIT* promoter hypermethylation, and stage IVA-B disease were selected as the reference group (HR = 1) in all study endpoints.
